# Cerebral Infarction in Immune Thrombotic Thrombocytopenic Purpura Is Associated with Old Age, Hypertension, Smoking, and Anti-ADAMTS13 Ig, But Not with Mortality

**DOI:** 10.1055/s-0040-1722610

**Published:** 2021-01-13

**Authors:** Raima Memon, Jingrui Sui, Chen Lin, X. Long Zheng

**Affiliations:** 1 Division of Laboratory Medicine, Department of Pathology, The University of Alabama at Birmingham, Birmingham, Alabama, United States; 2 Department of Neurology, The University of Alabama at Birmingham, Birmingham, Alabama, United States; 3 Department of Pathology and Laboratory Medicine, University of Kansas Medical Center, Kansas City, Kansas, United States

**Keywords:** thrombotic thrombocytopenic purpura, inflammatory mediators, ADAMS/ADAMTS13, von Willebrand factor, cerebrovascular disease, autoimmune diseases, autoantibodies

## Abstract

**Background** Neurological involvement is common in patients with immune thrombotic thrombocytopenic purpura (iTTP), but the frequency, risk factors, and outcomes of these with imaging-confirmed stroke in iTTP are not known.

**Methods** We selected 66 out of 109 iTTP patients with neurological signs and symptoms and reviewed their CT/MRI (computed tomography/magnetic resonance imaging) findings for the evidence of stroke and other clinical information in Alabama TTP Registry.

**Results** Of these, 52 (78.8%) had their CT/MRI done on admission in whom 22 (42.3%) were positive for multiple acute or chronic infarcts. The patients with image-confirmed ischemic stroke were older, and appeared to be associated with a history of hypertension and smoking. Additionally, patients with imaging-confirmed stroke showed higher plasma concentrations of anti-ADAMTS13 IgG than those without stroke. More interestingly, there was no statistically significant difference in the rate of exacerbation and 60-day mortality between those with and without stroke.

**Conclusion** Ischemic cerebral infarcts are common findings in brain imaging studies of patients with acute iTTP; old age, chronic hypertension, and smoking, as well as high plasma concentrations of anti-ADAMTS13 IgG may be the potential risk factors for cerebral infarction in these patients. The presence of image-confirmed ischemic stroke, however, does not predict exacerbation and 60-day mortality, although the long-term effect of such ischemic brain damage on cognitive function and quality of life remains to be determined.

## Introduction

Immune-mediated thrombotic thrombocytopenia purpura (iTTP) is a rare but potentially fatal blood disorder. It is characterized clinically by severe thrombocytopenia (platelet count, usually less than 30 × 10 ^9^ /L), microangiopathic hemolytic anemia (e.g., low hematocrit and hemoglobin, low haptoglobin, elevated lactate dehydrogenase (LDH), and presence of schistocytes, etc.), and organ injury. 1 2 The incidence of iTTP ranges from two to six cases per million residents, 3 4 but may be much higher in the African American population. 4 The iTTP patients may present with a thrombotic event, either initially or during the course of treatment, including ischemic cerebral infarction (i.e., stroke), 5 6 7 and acute myocardial infarction (MI). 8 9 10 The clinical presentation may range from mild nonspecific symptoms, including weakness, fatigue, headache, nausea and vomiting to stroke, MI and acute renal failure, and death. 11 12 The classic pentad with fever, thrombocytopenia, hemolytic anemia, neurological signs, and renal failure occurs in less than 10% of iTTP patients. 13 14 Thus, the presence of thrombocytopenia and microangiopathic hemolytic anemia is sufficient for the presumptive diagnosis of TTP. 15 16 17

To confirm the diagnosis of iTTP, plasma ADAMTS13 activity and inhibitors or anti-ADAMTS13 IgGs must be performed in a timely manner. 18 ADAMTS13 is a plasma metalloprotease that cleaves von Willebrand factor (VWF), thus regulating normal hemostasis. 19 20 21 If plasma ADAMTS13 is less than 10 U/dL (or 10% of normal), TTP diagnosis is confirmed; if plasma ADAMTS13 is greater than 20 U/dL (or 20% of normal), the TTP is essentially ruled out. If plasma ADAMTS13 activity is between 10 and 20 U/dL (or 10–20% of normal), clinical judgment is required to interpret this result. 18 Most adult TTP patients (greater than 95%) are caused by IgG type autoantibodies against ADAMTS13. 4 22 A positive inhibitor test (>0.4 U/mL) or an increased level of anti-ADAMTS13 IgG (>15 U/mL) is confirmatory for iTTP. 4 15 16 17 However, a negative inhibitor or antibody test does not automatically rule out the diagnosis of iTTP. Further follow-up tests and the observation of response to therapeutic plasma exchange (TPE) may provide additional information for the final diagnosis of iTTP.

Neurological signs and symptoms are common as the initial complaints in many patients with iTTP. 11 12 23 This may be resulted from the formation of microvascular thrombosis in small arteries and capillaries in the central nervous system, leading to transient (known as reversible encephalopathy) 24 25 or permanent ischemic brain damage. 5 6 7 These thrombi in large or small vessels in the brain may cause these neurological signs and symptoms ranging from headache, forgetfulness, numbness, and weakness of the extremities, and confusion to more severe forms such as seizure, stroke, coma, and death. Therefore, early recognition and prompt treatments such as TPE, 12 23 26 steroids, and caplacizumab (an anti-VWF nanobody) 27 28 29 are important as these microthrombi can lead to permanent brain damage leading to high morbidity and mortality. Rituximab (an anti-CD20 monoclonal antibody) helps suppress antibody production, thus reducing relapses in these patients. 30 31 32 33 34 35 36 37

Present study aims to determine the frequency and risk factors of ischemic stroke in patients with iTTP and the association of such an ischemic stroke with the exacerbation and 60-day mortality rate in these patients. The results of this study may help stratify patients for more aggressive treatment strategy, thus reducing long-term morbidity.

## Methods

*Patients:* The study was approved by the Institutional Review Board (IRB) of the University of Alabama at Birmingham, Alabama. The patient cohort from April 2006 and December 2019 was included for the study. All the patients were referred to us for treatment with TPE. Of 109 episodes, 66 unique patients with confirmed iTTP and presenting neurological symptoms at the time of the admission were identified for the study. The neurological symptoms range from mild complaints of headache, dizziness to severe complaints of slurring of speech, forgetfulness, visual changes, numbness, and weakness of the extremities or coma, and seizures.

Patient's clinical information was extracted from electronic medical records for patient demographics (e.g., age, sex, race, blood group) and presence of any comorbidities in these patients (e.g., hypertension, diabetes, history or current smoker, cancer, HIV, other autoimmune diseases, etc.). The data for these patients was further searched for management of these patients when presenting with these neurological symptoms and if neuroimaging studies (either computed tomography, CT or magnetic resonance imaging, MRI) were performed at the time of presentation for a suspected stroke or transient ischemic attack. For the patients who had CT/MRI done, results of the imaging were reviewed by a vascular neurologist to identify the presence or absence of confirmed stroke.

*Sample collection and laboratory testing:* All patients with confirmed iTTP included in this study had blood samples collected at the time of presentation or prior to initial TPE. Whole blood was drawn from an apheresis catheter and anticoagulated with 0.32% sodium citrate and centrifuged at 1,500 x *g* for 15 minutes. Plasma was separated from cellular components within 2 hours of collection and stored in aliquots at −80°C until testing.

The laboratory parameters, including complete blood count, white blood count, hematocrit, platelet count, creatinine, prothrombin time (PT), partial prothrombin time, fibrinogen, D-dimer, LDH, and troponin-I were all performed routinely for patient care and the data were extracted from electronic medical record or the Alabama Registry database.

ADAMTS13 activity and inhibitor titers were determined in the reference laboratory ( *Versiti* , Milwaukee, Wisconsin).

Anti-ADAMTS13 IgG was determined using a commercial ELISA kit (DiaPharma, West Chester, Ohio) according to the manufacturer's protocol. 38

Plasma histone-DNA complexes (Millipore Sigma), cell-free DNA (cfDNA) (Thermo Fisher Scientific, Waltham, Massachusetts), 4 and citrullinated histone H3 (Cayman chemicals, Ann Arbor, Michigan) were determined using commercially available reagents, according to the manufacturers' protocols.

*Statistical analysis:* A 60-day mortality and exacerbation-free survival rate were determined. Exacerbation was defined as the disease recurred within 30 days after the cessation of TPE. All statistical analyses were performed with the SPSS 26.0 software. For data with normal distribution, values were expressed as the mean ± standard deviation. For data that is not normally distributed, the values were expressed as the median and interquartile range. Student *t* -test was used to determine differences between two groups with normally distributed data, while the Mann-Whitney U test was used to analyze data that were not normally distributed. Log-rank test was used to compare the death-free survival rate between two groups. *p* -Values of less than 0.05 and less than 0.01 were considered statistically significant and highly statistically different, respectively.

## Results

*Patient characteristics* : Of 109 episodes with a confirmed diagnosis of iTTP (i.e., ADAMTS13 activity less than 10 IU/dL or 10% of normal, and inhibitor >0.4 U/mL or anti-ADAMTS13 IgG >15 U/mL), 66 (60.5%) presented with neurological signs and symptoms. Review of the patients' electronic medical record found that 14 (21.2%) of these patients with neurological complaints did not have neuroimaging study (CT/MRI) done during the entire hospitalization. However, 52 (78.8%) patients did have CT/MRI done, which was available for imaging review. Of these, 22 (33%) patients had confirmed stroke on CT/MRI, including both small and large vessels strokes, and 30 (45.4%) patients had no evidence of stroke on CT/MRI. For further analysis, the patients without any imaging and those with no evidence of stroke were combined into one group as “without stroke.”

*Association of patient demographics and comorbidities with stroke:* Of 66 patients presented with neurological signs and symptoms, the mean age was 45.2 years (standard deviation, SD of 13.7 years) at the time of presentation. The patients with imaging-confirmed stroke were older (the mean age, 48.7 years) as compared with those without stroke (the mean age, 43.4 years). There was no difference in gender as shown in Table 1 . However, of those with imaging-confirmed stroke, 13/22 (59.1%) were females. Of 66 patients in the study cohort, 49 (74.2%) were African American (AA), and of those 15 (68.2%) had imaging-confirmed stroke, significantly higher than that in all iTTP patients.

**Table 1:** Clinical characteristics in all iTTP patients with or without an imaging-confirmed ischemic stroke

Clinical information	Total *N* = 66	Without stroke ( *N* = 44)	Confirmed stroke ( *N* = 22)	*p* -Value
Age (year)	45.2 ± 13.7	43.4 ± 14.1	48.7 ± 12.3	0.142
Female, *n* (%)	33 (50)	20 (45.5)	13 (59.1)	0.434
African American, *n* (%)	49 (74.2)	34 (77.3)	15 (68.2)	0.309
Blood group O, *n* (%)	41 (62.1)	29 (65.9)	12 (54.5)	0.426
Initial episode, *n* (%)	14 (21.2)	6 (13.6)	8 (36.4)	0.054
Hypertension, *n* (%)	40 (60.6)	22 (50)	18 (81.8)	0.027 a
Diabetes mellitus, *n* (%)	16 (24.2)	13 (29.5)	3 (13.6)	0.226
Smoking ( *N* = 56), *n* (%)	27 (48.2)	14 (36.8)	13 (72.2)	0.021 a
SLE ( *N* = 38), *n* (%)	3 (7.9)	3 (13.6)	0 (0)	0.249

Abbreviations: iTTP, immune thrombotic thrombocytopenic purpura; *N* , total cases evaluated; *n* , the number of cases in each subgroup; SLE, systemic lupus erythematatosus.

a *p* < 0.05 indicates the statistical significance.

Only 14 (21.2%) patients were presenting as initial episode, and remainder were admitted as a relapse. Majority of the patients had one or more associated comorbidities, 40 (60.6%) had an associated hypertension ( *p* = 0 *.02* ). History of smoking was available in 56 patients. Of those, 27 (48.2%) of the patients had history of smoking or were current smokers. The association was statistically significant in patients with imaging confirmed stroke *(p = 0* .021). Only 16 (24.2%) of patients with neurological symptoms had diabetes mellitus and it was not statistically significant ( Table 1 ).

**Fig. 1:**
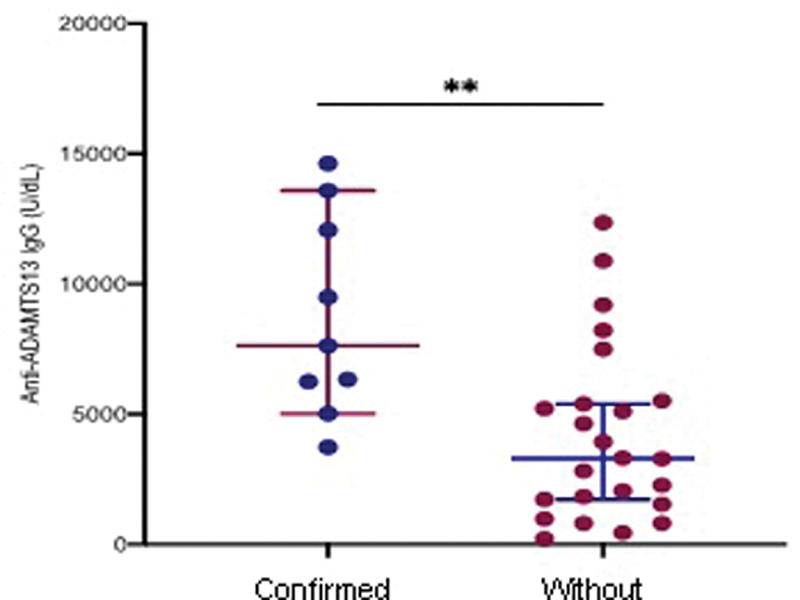
Plasma anti-ADAMTS13 IgG in patients with iTTP with ( **A** ) or without ( **B** ) imaging-confirmed stroke. Each dot represents each patient value; the horizontal bars inside dots are median and interquartile range (IQR). Mann-Whitney test was performed for statistical significance between two groups. *p* < 0.05 is statistically significant.

*Correlation of laboratory parameters in patients with stroke:* The routine laboratory parameters were consistent with the diagnosis of iTTP in all patients as shown in Table 2 . The mean platelet count in the patients was 12.8 × 10 9 /L, with no significant difference in between groups with and without imaging-confirmed stroke. The mean hemoglobin and hematocrit were 8.4 and 24.4%, respectively. PT, activated thromboplastin time, and serum fibrinogen were in normal range ( Table 2 ). Serum D-dimer was elevated in all patients with the mean value of 1,902.5 ng/mL, which was significantly increased in patients with imaging-confirmed stroke ( *p = 0* .046). All patients included for this cohort had confirmed diagnosis of iTTP with ADAMTS13 activity less than 10 U/dL or 10% of normal. ADAMTS13 inhibitors were available for only 36 patients with the mean value of 1.4 BU and there was no statistically significant difference between the two groups ( Table 3 ). Additionally, plasma levels of anti-ADAMTS13IgG were all above the normal range. The levels were significantly higher in patients with than those without an imaging-confirmed stroke ( *p* = 0.003) ( Fig. 1 ; Table 3 ).

**Table 2:** Routine laboratory parameters in iTTP patients with or without imaging-confirmed ischemic stroke

Laboratory parameters	All ( *N* = 66)	Without stroke ( *N* = 44)	Confirmed stroke ( *N* = 22)	*p* -Value
WBC (x 10 ^9^ /L), mean ± SD	10.8 (7.6–13.2)	11.1 (7.8–13.2)	8.7 (7.6–12.7)	0.297
Hb (g/dL), mean ± SD	8.4 ± 1.7	8.4 ± 1.6	8.5 ± 1.9	0.81
Hct (%), mean ± SD	24.4 ± 4.9	24.2 ± 4.7	24.3 ± 5.4	0.641
Platelet count (× 10 ^9^ /L), median (IQR)	12.8 (8.6–19.4)	14 (8.4–19.4)	12 (8.6–24.2)	0.948
Creatinine (mg/dL), median (IQR), *n* = 63	1.3 (0.9–1.7)	1.3 (1.0–1.6)	1.3 (0.9–1.8)	0.726
PT (s), median (IQR), *n* = 61	14.9 (14.1–15.9)	14.9 (14.3–15.7)	14.9 (14–16.1)	0.92
aPPT (s), mean ± SD, *n* = 58	31.7 ± 5.8	31.3 ± 508	32.4 ± 6.0	0.515
Fibrinogen (mg/dL), *n* = 55, mean ± SD	401.3 ± 131.9	385.5 ± 139.4	431.3 ± 113.8	0.224
D-dimer (ng/dL), median (IQR), *n* = 63	1,902.5 (1,086.3–5,195.8)	2,631 (1,370–5,804)	1,138 (715.5–2,498)	0.046 a
Troponin-I (ng/dL), median (IQR)	0.5 (0.1–1.3)	0.5 (0.1–1.2)	0.7 (0.2–2.8)	0.273

Abbreviations: aPTT, activated thromboplastin time; Hct, hematocrit; IQR, interquartile range; iTTP, immune thrombotic thrombocytopenic purpura; *N* , total number of cases; *n* , number of cases in this category; PT, prothrombin time; WBC, white blood cells.

a *p* -Value < 0.05 indicates statistical significance.

**Table 3:** Special laboratory parameters in iTTP patients with or without imaging-confirmed ischemic stroke

	All cases ( *N* = 66)	Without stroke ( *N* = 44)	Confirmed stroke ( *N* = 22)	*p* -Value
Anti-ADAMTS13 IgG (U/mL), *n* = 36	5,022 (1,954–7,929)	3,307 (1,588–5,486)	7,634 (5,638–1,2830)	**0.003** a
Inhibitor (U/mL), *n* = 64	1.4 (0.5–4.6)	1.4 (0.5–4.0)	1.4 (0.5–5.6)	0.92
CitH3 (ng/mL), *n* = 45)	4.4 (2.5–7.0)	4.9 (2.1–7.2)	3.5 (2.6–7.1)	0.758
Histone/DNA complexes (U/mL), *n* = 42)	69.3 (37.1–144.4)	69.2 (38.0–142.2)	80.6 (33.6–160.4)	0.703
cfDNA (ng/mL), *n* = 48)	983 (812–1,286)	1,040 (824–1,701)	913 (791–1,122)	0.616

Abbreviations: *N* , total number of cases; *n* , number of cases assayed for this biomarker.

a *p* -Value < 0.01 indicates that the difference between two groups is statistically highly significant.

**Fig. 2:**
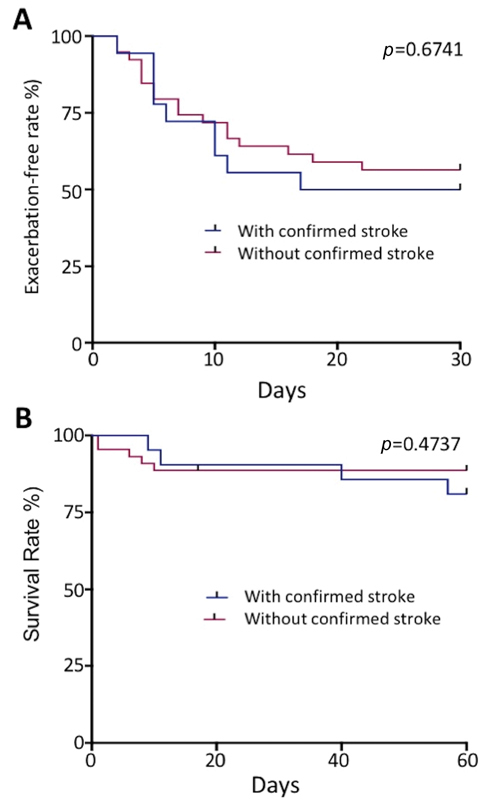
Kaplan-Meier survival analysis in patients with or without stroke *.* ( **A** ) Exacerbation free rate (%) in patients with or without stroke 30 days following the cessation of TPE; ( **B** ) 60-day survival rate in patients with or without stroke since admission.

To assess if acute inflammation, neutrophil activation, and NETosis play a role in pathogenesis of stroke, we determined plasma NETosis markers including histone/DNA complexes, citrH3, and cfDNA in these patients. To our surprise, while plasma levels of histone/DNA complexes, citH3, and cfDNA were dramatically elevated in all patients with acute iTTP, their levels in iTTP patients with stroke were not significantly higher than those without stroke ( Table 3 ).

*Association of stroke with mortality and exacerbation:* The 60-day mortality and exacerbation-free survival were determined in all patients. Using the log-rank test, we found no association of 60-day mortality and exacerbation-free survival rate with stroke. As shown in Fig. 2 , Kaplan-Meier survival analysis demonstrated that there was no statistically significant difference in 60-day mortality rate and exacerbation-free survival rate between patients with and without imaging-confirmed stroke, suggesting that ischemic stroke does not adversely affect patient's survival or predicts exacerbation during acute episode while the long-term impact on the brain function is not known.

## Discussion

In the present study, we report the clinical and laboratory characteristics of 66 patients with acute iTTP who presented with neurological signs and symptoms in a single institution. These patients are divided into two groups, based on the presence or the absence of cerebral infarction on the neuroimaging study. We found that older age, black race, and comorbidities such as cardiovascular disease, diabetes, and smoking, are associated with ischemic cerebral infarction. So were plasma levels of anti-ADAMTS13 IgG, but not the inflammatory or NETosis markers, 60-day mortality, and exacerbation.

Most published studies to date are either a case report or small series of report that addressed the stroke in patients with acute TTP. 5 6 7 Burrus et al reported 46 patients with TTP who had neuroimaging done. 24 No association of imaging-confirmed stroke with comorbidities was found. In our study, more than half of the iTTP patients presented with one or more neurological sign or symptoms as their initial complaint on admission. The symptoms range from headache, dizziness, numbness and weakness in the extremities, confusion to loss of consciousness, visual changes, disorientation, altered mental status, and generalized seizures. In our cohort, no patient presented with headache as the sole neurological symptom. Surprisingly, in two-third cases in which neuroimaging was performed approximately 42.5% had imaging-confirmed small and large vessel stroke. There was no significant difference in gender of patients presenting with neurological symptoms. However, there was female predominance in patient with imaging-confirmed stroke. Patients with imaging confirmed stroke were older than those without stroke. As previously reported, majority of our such patients were African Americans, 4 12 and also majority of the patients with neurological signs and symptoms were presenting with a relapse disease or sometime during remission (when platelet count was normal). Black is shown to be at risk for the development of autoantibody against ADAMTS13 and mortality, 39 resulting from lack of the protective HLA-DRB1*04. 40

Analyzing other risk factors in these patients, all patients with imaging-confirmed stroke showed a significant association with hypertension and smoking. This may not be the surprising findings as the association of hypertension with vascular damage and thromboembolic events in TTP patients has been well established. 41 42 However, the association with smoking in iTTP patients with stroke has not been previously observed. In view of the clinical manifestations of the iTTP that often involve serious consequences, such as thrombotic events, it is necessary to know other factors and conditions that may result in a worse outcome. 43 44 There was no significant association of iTTP stroke with diabetes in our study.

The D-dimer was significantly increased in all patients diagnosed with acute iTTP, which was strongly correlated with an imaging-confirmed stroke. Consistent with those previously reported, the high levels of anti-ADAMTS13 IgG correlate with disease progression and potential relapse in patients with acute iTTP. 38 45 However, the strong association between anti-ADAMTS13 IgG levels (but not inhibitor titer) and stroke had not previously observed. To our surprise, there was no association between plasma levels of histone/DNA complexes, citH3, and cfDNA and imaging confirmed stroke, despite a marked elevation of these markers in patients with acute iTTP compared with the healthy controls or in patients during remission. 4 46 47 49 These inflammatory and NETosis markers appear to be associated with the size of ischemic stroke 48 and in-hospital mortality. 49

We recognize the potential limitations in the present study. First, not all iTTP patients had neuroimaging done on admission and during hospitalization; second, some of the clinical and imaging information were obtained retrospectively, which may not be complete; third, the number of cases in both groups were relatively small due to the rarity of iTTP. This may have a low statistical power to detect the difference in exacerbation or mortality rate in patients with or without stroke.

In conclusion, our present study demonstrates a proof of concept that patients with acute iTTP who present with neurological signs and symptoms may have a higher incidence of ischemic stroke than we have previously known. The risk factors for developing such an ischemic stroke in iTTP patients include older age, comorbid hypertension, and smoking; additionally, high admission plasma levels of anti-ADAMTS13 IgG, but not inflammatory/NETosis markers, are associated with ischemic stroke. To our surprise, no correlation between the ischemic stroke and iTTP recurrence or mortality was identified, although the long-term neurological sequelae of stroke were yet to be assessed. We propose that a prospective and longitudinal neuroimaging study for patients with iTTP should be conducted to assess the incidence rate, risk factors, reversibility, and long-term outcomes associated with ischemic stroke.
